# Multimorbidity resilience and COVID-19 pandemic self-reported impact and worry among older adults: a study based on the Canadian Longitudinal Study on Aging (CLSA)

**DOI:** 10.1186/s12877-022-02769-2

**Published:** 2022-02-02

**Authors:** Andrew Wister, Lun Li, Theodore D. Cosco, Jacqueline McMillan, Lauren E. Griffith, on behalf of the Canadian Longitudinal Study on Aging (CLSA) Team, Andrew Costa, Laura Anderson, Cynthia Balion, Susan Kirkland, Asada Yukiko, Christina Wolfson, Nicole Basta, Benoȋt Cossette, Melanie Levasseur, Scott Hofer, Theone Paterson, David Hogan, Teresa Liu-Ambrose, Verena Menec, Philip St. John, Gerald Mugford, Zhiwei Gao, Vanessa Taler, Patrick Davidson, Paminder Raina

**Affiliations:** 1 Gerontology Research Centre & Department of Gerontology, Simon Fraser University, 2800-515 Hastings Street, Vancouver, BC V6B 5K3 Canada; 2 Gerontology Research Centre, Simon Fraser University, 2800-515 Hastings Street, Vancouver, BC V6B 5K3 Canada; 3 Oxford Institute of Population Ageing, University of Oxford, 66 Banbury Road, Oxford, OX2 6PR UK; 4 Department of Medicine, Section of Geriatric Medicine, University of Calgary, Calgary, AB Canada; 5 O’Brien Institute for Public Health, University of Calgary, Calgary, AB Canada; 6 Labarge Centre for Mobility in Aging, McMaster University, Hamilton, ON Canada; 7 McMaster Institute for Research on Aging, McMaster University, Hamilton, ON Canada; 8 Department of Health Research Methods, Evidence, and Impact, Faculty of Health Sciences, McMaster University, Hamilton, ON Canada

**Keywords:** Resilience, Multimorbidity, COVID-19, Older adults, CLSA

## Abstract

**Background:**

The Coronavirus Disease-2019 (COVID-19) pandemic has created a spectrum of adversities that have affected older adults disproportionately. This paper examines older adults with multimorbidity using longitudinal data to ascertain why some of these vulnerable individuals coped with pandemic-induced risk and stressors better than others – termed multimorbidity resilience. We investigate pre-pandemic levels of functional, social and psychological forms of resilience among this sub-population of at-risk individuals on two outcomes – self-reported comprehensive pandemic impact and personal worry.

**Methods:**

This study was conducted using Follow-up 1 data from the Canadian Longitudinal Study on Aging (CLSA), and the Baseline and Exit COVID-19 study, conducted between April and December in 2020. A final sub-group of 9211 older adults with two or more chronic health conditions were selected for analyses. Logistic regression and Generalized Linear Mixed Models were employed to test hypotheses between a multimorbidity resilience index and its three sub-indices measured using pre-pandemic Follow-up 1 data and the outcomes, including covariates.

**Results:**

The multimorbidity resilience index was inversely associated with pandemic comprehensive impact at both COVID-19 Baseline wave (OR = 0.83, *p* < 0.001, 95% CI: [0.80,0.86]), and Exit wave (OR = 0.84, *p* < 0.001, 95% CI: [0.81,0.87]); and for personal worry at Exit (OR = 0.89, p < 0.001, 95% CI: [0.86,0.93]), in the final models with all covariates. The full index was also associated with comprehensive impact between the COVID waves (estimate = − 0.19, *p* < 0.001, 95% CI: [− 0.22, − 0.16]). Only the psychological resilience sub-index was inversely associated with comprehensive impact at both Baseline (OR = 0.89, *p* < 0.001, 95% CI: [0.87,0.91]) and Exit waves (OR = 0.89, p < 0.001, 95% CI: [0.87,0.91]), in the final model; and between these COVID waves (estimate = − 0.11, p < 0.001, 95% CI: [− 0.13, − 0.10]). The social resilience sub-index exhibited a weak positive association (OR = 1.04, *p* < 0.05, 95% CI: [1.01,1.07]) with personal worry, and the functional resilience measure was not associated with either outcome.

**Conclusions:**

The findings show that psychological resilience is most pronounced in protecting against pandemic comprehensive impact and personal worry. In addition, several covariates were also associated with the outcomes. The findings are discussed in terms of developing or retrofitting innovative approaches to proactive coping among multimorbid older adults during both pre-pandemic and peri-pandemic periods.

**Supplementary Information:**

The online version contains supplementary material available at 10.1186/s12877-022-02769-2.

## Background

Older adults have been disproportionately affected by the new coronavirus disease 2019 (COVID-19) pandemic due to the intersections of intensified fear of susceptibility and seriousness of infection, coupled with higher-than-average infection rates and high mortality [1–3]. Negative effects of social/physical distancing policies have been associated with adverse health, psychological, social and economic consequences of the pandemic [4–9]. Furthermore, it has been shown that individuals of advanced age will likely experience greater challenges during the recovery phase of the pandemic due to their more complex health statuses and system disruptions to community and healthcare arenas [10, 11]. However, some individuals may possess important protective factors, resources and strengths that enable them to cope better than others during the pandemic [12–17]. For instance, in a sample of older adults, Igarashi et al. found that 93% described experiencing vulnerabilities directly linked to the pandemic; yet, about two-thirds identified positive responses to these adversities, what they concluded represented a form of resilience [13]. Those with multimorbidity are particularly prone to greater challenges during the pandemic, since multimorbidity adversely affects older adults on many levels, including physiological, functional, psychological and social ones [1–3, 18]. The aim of this paper is to explore whether and to what degree different forms of resilience influence perceptions of coping during the pandemic among persons living in the community with multiple chronic conditions.

Multimorbidity is present when an individual has been diagnosed with two or more concurrent chronic diseases - a condition that is slow in progression, long in duration, typically limiting function, productivity, system burden and quality of life [19]. Approximately two-thirds of older adults have multimorbidity in most countries with developed health care systems [20, 21]. It has been established that older adults with pre-existing common physical conditions (e.g., cardiovascular disease, cancer, diabetes, obesity, and chronic obstructive pulmonary disease), as well as pre-existing mental conditions, are at heightened pandemic risk sequalae on quality of life, including psychological well-being, depression, distress and anxiety, and social isolation [1–3, 7, 14, 18]. Further, during the pandemic, there has been an exacerbation of mental health adversity among multimorbid older adults due to physical distancing mitigation policies, as well as general impact on life, requiring further research into forms of resilience [8, 16, 22].

To date, early COVID-19 pandemic research has provided inconsistent evidence of the key protective and coping processes among older persons generally, and especially among those with multimorbidity [5, 15]. Previous research into resilience underscores the need to consider multi-level integrated domains that connect individual and system-level responses [23]. Thus, extending our understanding of these processes in older age from pre-pandemic to peri-pandemic will help to develop national and international efforts to support healthy ageing, such as the WHO initiative *Building Resilience: A Key Pillar of Health 2020 and the Sustainable Development Goals* [24]. The CLSA platform provides longitudinal data that can be used to measure and investigate resilience processes from pre-pandemic to peri-pandemic [25, 26].

### Conceptualizing pandemic multimorbidity resilience and aging

Recently, there have been model developments aimed at understanding how individuals respond to illness-related adversities and regain a sense of wellness in their lives, what can be termed multimorbidity resilience (MR) – the ability and resources needed to adapt and navigate illness adversity and its often stress-inducing experiences [27–31]. This form of adversity is challenging because those with multimorbidity, especially in old age, face interrelated disability episodes, daily and intermittent stressors, and often long-lasting impediments. The increased stressors associated with the COVID-19 pandemic raises questions about patterns of resilience [8, 16].

Although there are many resilience models, we employ the *Lifecourse Model of Multimorbidity Resilience* (LMMR) [30], which captures a complex and dynamic set of protective/risk traits, resources, and processes that occur over the lifecourse of the individual applied specifically to fostering resilience among older persons with multimorbidity. The present research focuses explicitly on three multimorbidity resilience domains (functional, social and psychological) conceptualized as combinations of both stress-inducing and stress-protecting measures. First, *functional multimorbidity resilience* is needed to maintain social roles and health promoting activity levels deemed to be fundamental to aging well with multimorbidity [32, 33]. Second, successful activation of *social multimorbidity resilience* entails harnessing available resources in the social network that may assist in coping responses [27]. While social multimorbidity resilience has been associated with higher levels of health behaviours among multimorbid older adults [34], the restrictions on social contact during the pandemic may actually result in lower perceived impact and worry, since those with larger social networks pre-pandemic may have had more to lose. Finally, drawing from stress theory and the cognitive appraisal process [35, 36], *psychological multimorbidity resilience* fosters better coping with multimorbidity. We expect that this form of resilience will have the strongest associations with pandemic impact and worry among older adults with multimorbidity.

### Research gaps, aims, and hypotheses

Formative research has offered some evidence that resilience is critical for coping with and navigating the stressful situations generated by the pandemic [6, 8, 13, 22]. Grossman et al. provided evidence for the moderating effect of resilience between COVID-19-related loneliness and sleep problems among older adults [6]. Another study using mental distress as an indicator of psychological resilience during the initial outbreak of the pandemic reported that low and normal resilience groups experienced increases in mental distress compared to adults with high resilience [22]. Research by Pearman et al. and Whitehead suggests that older persons who engaged in proactive coping at the start of the pandemic were able to reduce the level of pandemic stress and maximize psychological well-being, used as an indicator of resilience [15, 16]. Yet, we know little about this type of coping among older adults with multimorbidity [14], or whether the impact of the pandemic leveled or declined during different time periods [4, 37].

The proposed study employs pre- and peri-pandemic CLSA data waves to test three hypotheses: 1) Total MR index (MRI) will be inversely associated with self-reported overall (comprehensive) impact of the pandemic as perceived by older adults. 2) Total MRI will be inversely associated with self-reported worry about COVID-19. 3) Of the three multimorbidity sub-indices, psychological resilience will be more strongly associated with pandemic impact and worry than functional and social sub-indices. All hypotheses will include covariates of MRI drawn from the broader literature on multimorbidity and aging [19, 21, 38, 39]. These include a spectrum of socio-demographic and mutable factors, including age, sex, marital status, visible minority and immigration status, socio-economic status, living/housing environment, etc.

## Methods

### Data and sample

This study utilized the second wave (Follow-up 1) of the main Canadian Longitudinal Study on Aging (CLSA), and the COVID Baseline (COVID-B) and Exit (COVID-E) survey waves. The CLSA is a national-level population-based longitudinal study with an original baseline sample of 51,338 participants aged between 45 and 85 years when recruited between 2011 and 2015. Two cohorts form the CLSA participants, including the Comprehensive cohort who were randomly selected from the population within 25 km (50 km for lower population density area) of the 11 data collection sites across Canada, and the Tracking cohort randomly selected from the ten provinces of Canada through telephone interview. The Follow-up 1 wave was collected between 2015 and 2018 and included 44,817 participants from Baseline. A total of 28,559 CLSA participants took part in the CLSA COVID-B study (April–May, 2020) after the outbreak of the pandemic, with 24,114 participating in the Exit survey (September–December, 2020). Among participants unable to accept the invitation to take part in the COVID survey, 2500 had died, 3406 withdrew from the CLSA study, 2414 had outdated contact information or did not participate due to other administrative reasons, and 318 required a proxy to participate in the study deeming them ineligible. Data were collected either by email via a web questionnaire (*n* = 34,498) or by telephone (*n* = 8202).

In order to measure functional multimorbidity resilience, only participants who have been in the Comprehensive cohort, who were 65 and over at the time of the Follow-up 1 survey, and only participants self-reporting multimorbidity were included, resulting in a final sub-sample of 9211. Multimorbidity was defined as participants diagnosed with two or more of the following 27 chronic conditions collected in the CLSA Follow-up 1 wave (Alzheimer’s disease, back problems, bowel incontinence, cancer, cataracts, diabetes, epilepsy, glaucoma, heart attack, heart disease, high blood pressure, irritable bowel syndrome, kidney disease, Parkinson’s disease, peripheral vascular disease, lung disease, macular degeneration, multiple sclerosis, osteoarthritis, osteoporosis, migraine headaches, rheumatoid arthritis, stroke, thyroid problem, transient ischemic attack, ulcer, and urinary incontinence). Since the full set of chronic conditions were not collected during the COVID-19 surveys, we used Follow-up 1 data to identify the target group.

Detailed information about the CLSA has been published elsewhere [25, 26, 40]. Researchers can access the de-identified data, and information on weighting through the CLSA website (www.clsa-elcv.ca).

### Measurement

#### Outcome variables

There are two outcome variables: 1) self-reported comprehensive impact of the COVID-19 pandemic (CI), and 2) self-reported personal worry about COVID-19 (PW). Based on a similar measure developed by Cao-Lei and colleagues to measure cognitive appraisals of stressful Impact during other disasters, CI uses responses to the question: “Taking everything about COVID-19 into account, how would you describe the consequences of COVID-19 on you and your household?” [41]. The answers to this question include: “1 = very negative”, “2 = negative”, “3 = no effect”, “4 = positive”, and “5 = very positive,” Due to the ordinal nature of the variable and a positive skew in the distribution, we dichotomized the answers into two levels, including “1 = negative impact” (1 and 2) and “0 = positive/no impact” (3 to 5). CI was measured in an identical way at both the COVID-B and COVID-E waves. PW was collected only in the COVID-E wave, based on the question “How worried are you personally about COVID-19 at present” with a 7-point Likert scale, where “1= not at all worried,” and “7= very worried. We dichotomized this variable into “0=less worried” (1 to 4), and “1 = more worried” (5 to 7).

#### Independent variables

The primary independent variable is the multimorbidity resilience index (MRI). The MRI was developed using CLSA baseline data and has been shown to have good concurrent validity, having statistically significant positive associations with higher levels of perceived health and sleep quality, less perceived pain, and fewer hospital over-night stays and emergency department visits [31]. The MR measure contains three primary resilience sub-indexes), each of which is measured using three variables capturing positive and negative attribution. The MR functional sub-index (MR-FI) consists of the Older Americans Resources and Services Activities of Daily Living (ADL) Scale, Instrumental Activities of Daily Living (IADL) Scale [42] and Summary Performance Score of Functional Ability Scale [43]. The MR psychological sub-index (MR-PI) consists of the Kessler Psychological Distress K10 Scale [44], Center for Epidemiological Studies Depression (CES-D) Scale [45], and the Diener Satisfaction with Life Scale [46]. The MR social sub-index (MR-SI) consists of the total Medical Outcomes Study (MOS) Social Support Survey score [47], a social participation measure including the frequency of participation in activities with family and friends, and a single item measuring perceived loneliness over the past week (from the CES-D 10). In order to standardize the different measurement levels and ranges, a mapping system was applied to convert all the variables into a score between 0 to 10 [31]. The scores of each of the three index composite measures were summed and divided by three to produce a standard set of sub-index scores (range 0–10). A higher score is interpreted as higher levels of multimorbidity resilience. The total MRI score was calculated by adding the three sub-index scores and dividing by three to produce a measure with the same range (0–10). The correlations among the sub-indexes ranged between approximately .20 (functional and social domains) and .47 (psychological and social domains), indicating that they measure distinct resilience domains (See Supplementary Table 1). The MRI was calculated using the CLSA Follow-up 1 wave to measure pre-pandemic levels.

#### Covariates

Ten demographic and socio-economic social determinants of health were included in the data analysis [21]. Four fixed variables were extracted from the CLSA Baseline wave, including: sex, country of birth, ethnic status and highest educational attainment (which changed very little over time). Sex was categorized as “female” and “male”. Country of birth was categorized into two groups, including “born in Canada” and “out of Canada.” We used visible minority status as a crude indicator of cultural background: “visible minority” and “non-visible minority” (i.e., Caucasian). Education was regrouped from the original seven categories into three (due to small cases in some categories), including: “no post-secondary education,” “some post-secondary education (diploma and certificate),” and “university degrees (Bachelor and above).” Two covariates were extracted from the CLSA Follow-up 1 wave: marital status and income. Marital status was dichotomized into: “not married (single, widowed or separated),” and “married or in common-law relationship.” Personal income was initially measured at five levels, including less than $20,000, $20,000 to $49,999, $50,000 to $99,999, $100,000 to $149,999, and $150,000 and more, and the last two categories were regrouped into one due to small numbers. The remaining four covariates (age, household size, working status, living area) were measured using the CLSA COVID-B wave. Age was measured using single years of age, which we divided into young-old and old-old categories for comparisons: “65 to 74 years old” and “75 years and older.” Household size was measured by three levels, including: “1 person”, “2 persons”, and “3 persons or more”. Work status was measured as “working” or “non-working” (retired and those not in workforce for other reasons). Finally, rural/urban status was dichotomized as: “rural area” and “urban area.”

### Analytic procedure

SPSSX Version 26 was used for all analyses. As an initial step, we examined descriptive patterns in the data for all variables among older adults with multimorbidity by sex (female and male) and age groups (65 to 74 years old and 75 years and older). Sex and age have been identified as important correlates of multimorbidity [19]. Additional bivariate analyses were performed for the CI (COVID-B and COVID-E waves) and PW (COVID-E) based on the demographic and social-economic statuses. Subsequently, two sets of multivariable analyses were performed to examine the relationship between pre-pandemic MR index (and the three sub-indexes, MR-functional sub-index, MR-psychological sub-index, MR-social sub-index) on CI and PW between -B and COVID E waves. First, three sets of logistic regression analyses were conducted to examine the association between MRI scores (Total MRI score and three sub-index scores) and the two outcome variables: CI (COVID-B wave and COVID-E wave separately), and PW (COVID- E wave only). Two models were built for each set of logistic regressions. In the first unadjusted model, only the MRI and sub-index scores were included. In the second adjusted model, all ten demographic and socio-economic covariates were incorporated into the model. The odds ratios [EXP(B)], where EXP = exponential, and B = coefficient for each variable were reported. We also report the model fit in each table using two statistical indicators [− 2 Log Likelihood and Cox & Snell R^2^], where R^2^ = pseudo variance explained.

Additionally, since CI was measured at two points in the pandemic, we also performed Generalized Linear Mixed Models (GLMM) [48]. GLMM is specifically developed to conduct longitudinal repeated measure analysis, in this case to examine the comprehensive impact and worry, as well as the change from COVID-19 study Baseline to Exit survey. GLMM adjusts models for the random effects of repeated measuring on the same participants and can estimate both within- and between-subject variability. GLMM is suitable for examining dichotomous outcome variables as used in this study. To account for change in CI between COVID-B to COVID-E waves, the survey wave was included in the model as a covariate. Also, interactive terms between sex and survey wave, as well as age group and survey wave, were included in the models to further explore the associations revealed in the descriptive analyses. Similar to the logistic regression, two models were built for each set of GLMM. In the first model, only the survey wave and the MRI score were included. In the second model, the ten demographic and socio-economic covariates were added. The odds ratios [EXP(B)] were reported for studied variables. The model fit is indicated by the Akaike Information Criterion (AIC) [48], and a lower number indicates a better model fit.

Missing cases were examined through the Little’s MCAR test, indicating the missing pattern was random (*p* > 0.05). List-wise deletion for all variables was applied during data analyses. We conducted supplementary analyses with the missing values imputed according to the levels of measurement, which replicated the results; therefore, we report the findings based on the untreated data.

## Results

### Descriptive results

Among the 9211 older adults with multimorbidity, female participants constituted a slightly higher proportion (*n* = 5004, 54%) than males, and there were slightly more multimorbid participants aged between 65 and 74 years than those who were aged 75 years and older (*n* = 4835, 52% and *n* = 4376, 48% respectively). Most of the selected participants were married or in common-law relationship (*n* = 5958, 65%), living with one or more persons (*n* = 6146, 60%), educated with university degrees (*n* = 4174, 45%), non-working (*n* = 8087, 91%), receiving a moderate level of income ($20,000 to $49,999, *n* = 3805, 44%), living in an urban area (*n* = 8343, 91%), born in Canada (*n* = 7409, 80%) and Caucasian (*n* = 8873, 96%). In addition, slightly more participants were less worried (*n* = 4095, 52%) than more worried about the COVID-19 pandemic, and more than three-fifth of participants believed they and their family were negatively impacted by the COVID-19 pandemic at both Baseline and Exit waves (*n* = 5205, 62% and *n* = 4887, 64% respectively).

Female participants reported a lower total MR score (t_(7583)_ = − 11.30, *p* < 0.001), as well as for all three sub-index scores (t_(7561)_ = − 13.32, *p* < 0.001 for functional, t_(8708)_ = − 9.74, *p* < 0.001 for psychological and t_(8714)_ = − 5.95, *p* < 0.001 for social resilience, respectively) when compared to male participants. Also, a higher proportion of older female participants were more worried about the COVID-19 pandemic than males (49 and 46% respectively, χ ^2^(1) = 6.52, *p* < 0.05). In addition, a lower proportion of female participants were negatively affected by the COVID-19 pandemic than their male counterparts (59 and 65% respectively, χ ^2^(1) = 27.51, *p* < 0.001) at the Baseline wave. When it comes to the Exit wave, no statistical difference exists between male and female participants regarding comprehensive impact (see Table 1). When comparing to participants aged 75 years and older, the younger age group (65 to 74 years old) reported higher MRI scores (t_(7604)_ = 8.88, *p* < 0.001), MR-FI and MR-SI sub-index scores (t_(7375)_ = 23.69, *p* < 0.001, and t_(8872)_ = 7.33, *p* < 0.001 respectively), but lower MR-PI sub-index score (t_(8871)_ = − 2.18, *p* < 0.05). Also, a higher proportion of the younger age group were more worried about the COVID-19 pandemic (52 and 43% respectively, χ ^2^(1) = 60.53, *p* < 0.001). And a higher proportion of the younger age group were negatively affected by the COVID-19 pandemic than the older age group (65 and 63% respectively, χ ^2^(1) = 0.38, *p* < 0.05) at the Exit wave, but not the Baseline wave. For further detailed information regarding sex and age differences see Tables 1 and 2.

**Table 1:** Descriptive data showing all variables by sex (*n* = 9211)

	All participants *N* = 9211 n/% or mean(SD)	Female participants *n* = 5004 n/% or mean(SD)	Male participants *n* = 4207 n/% or mean(SD)	χ ^2^(df)/t test (df)
**Sex**		–	–	
Female	5004/ 54.33			
Male	4207/ 45.67			
**Age**				4.27(1) *
65 to 74 years old	4835/ 52.49	2676/ 53.48	2159/ 51.32	
75 years and older	4376/ 47.51	2328/ 46.52	2048/ 48.68	
**Marital status**				676.50 (1) ***
Not married	3239/ 35.18	2353/ 47.05	886/ 21.07	
Married / Common law	5968/ 64.82	2648/ 52.95	3320/ 78.93	
**Household size**				528.02 (2) ***
1 person	2795/ 31.26	2017/ 41.57	778/ 19.03	
2 persons	5178/ 57.91	2363/ 48.70	2815/ 68.84	
3 persons and more	968/ 10.83	472/ 9.73	496/ 12.13	
**Highest education**				200.24 (2) ***
No post-secondary education	2194/ 23.84	1333/ 26.67	861/ 20.49	
Post-secondary education	2834/ 30.80	1735/ 34.71	1099/ 26.15	
University degrees	4174/ 45.36	1931/ 38.63	2243/ 53.37	
**Work status**				42.28 (1) ***
Work	841/ 9.42	369/ 7.58	472/ 11.62	
Non-working	8087/ 90.58	4497/ 92.42	3590/ 88.38	
**Personal income**				725.12 (3) ***
Less than $20,000	1186/ 13.61	937/ 20.11	249/ 6.15	
$20,000 to $49,999	3805/ 43.68	2251/ 48.30	1554/ 38.35	
$50,000 to $99,999	2895/ 33.23	1255/ 26.93	1640/ 40.47	
$100,000 and more	826/ 9.48	217/ 4.66	609/ 15.03	
**Living area**				0.01 (1)
Rural area	824/ 8.99	446/ 8.96	378/ 9.02	
Urban area	8343/ 91.01	4531/ 91.04	3812/ 90.98	
**Country of birth**				23.51 (1) ***
Canada	7409/ 80.44	4117/ 82.27	3292/ 78.25	
Out of Canada	1802/ 19.56	887/ 17.73	915/ 21.75	
**Ethnicity status**				6.02 (1) *
Not visible minority	8873/ 96.42	4843/ 96.86	4030/ 95.91	
Visible minority	329/ 3.58	157/ 3.14	172/ 4.09	
**Total resilience score**	6.45 (1.62)	6.25 (1.68)	6.66 (1.52)	−11.30 (7583) ***
**Functional resilience score**	7.23 (1.87	6.97 (2.09)	7.51 (1.55)	−13.32 (7561) ***
**Psychological resilience score**	5.18 (2.89)	4.91 (2.89)	5.50 (2.85)	−9.74 (8708) ***
**Social resilience score**	6.71 (1.90)	6.60 (1.92)	6.84 (1.87)	−5.95 (8714) ***
**Personal worry at Baseline wave**				6.52 (1) *
Less worry	4095/ 52.09	2191/ 50.79	1904/ 53.68	
More worry	3766/ 47.91	2123/ 49.21	1643/ 46.32	
**Comprehensive impact at Baseline wave**				27.51 (1) ***
Positive	3244/ 38.40	1857/ 40.98	1387/ 35.41	
Negative	5205/ 61.60	2675/ 59.02	2530/ 64.59	
**Comprehensive impact at Exit wave**				0.34 (1)
Positive	2718/ 35.74	1465/ 35.45	1253/ 36.09	
Negative	4887/ 64.26	2668/ 64.55	2219/ 63.91	

Note: * *p* < 0.05, **, *p* < 0.01, ***, *p* < .001

**Table 2:** Descriptive data showing all variables by age groups (*n* = 9211)

	All participants *N* = 9211 n/% or mean(SD)	65 to 74 years old participants *n* = 4835 n/% or mean(SD)	75 years and older participants *n* = 4376 n/% or mean(SD)	χ ^2^(df)/t test (df)
**Sex**				4.27(1) *
Female	5004/ 54.33	2676/ 55.35	2328/ 53.20	
Male	4207/ 45.67	2159/ 44.65	2048/ 46.80	
**Age**		–	–	
65 to 74 years old	4835/ 52.49			
75 years and older	4376/ 47.51			
**Marital status**				118.03 (1) ***
Not married	3239/ 35.18	1452/ 30.04	1787/ 40.86	
Married / Common law	5968/ 64.82	3382/ 69.96	2586/ 59.14	
**Household size**				210.76 (2) ***
1 person	2795/ 31.26	1186/ 25.06	1609/ 38.23	
2 persons	5178/ 57.91	2908/ 61.45	2270/ 53.93	
3 persons and more	968/ 10.83	638/ 13.48	330/ 7.84	
**Highest education**				73.91 (2) ***
No post-secondary education	2194/ 23.84	979/ 20.26	1215/ 27.80	
Post-secondary education	2834/ 30.80	1529/ 31.64	1305/ 29.86	
University degrees	4174/ 45.36	2324/ 48.10	1850/ 42.33	
**Work status**				335.09 (1) ***
Work	841/ 9.42	697/ 14.76	144/ 3.42	
Non-working	8087/ 90.58	4025/ 85.24	4062/ 96.58	
**Personal income**				30.42 (3) ***
Less than $20,000	1186/ 13.61	654/ 14.08	532/ 13.08	
$20,000 to $49,999	3805/ 43.68	1925/ 41.44	1880/ 46.23	
$50,000 to $99,999	2895/ 33.23	1567/ 33.74	1328/ 32.65	
$100,000 and more	826/ 9.48	499/ 10.74	327/ 8.04	
**Living area**				32.77 (1) ***
Rural area	824/ 8.99	511/ 10.61	313/ 7.19	
Urban area	8343/ 91.01	4303/ 89.39	4040/ 92.81	
**Country of birth**				59.70 (1) ***
Canada	7409/ 80.44	4036/ 83.47	3373/ 77.08	
Out of Canada	1802/ 19.56	799/ 16.53	1003/ 22.92	
**Ethnicity status**				0.10 (1)
Not visible minority	8873/ 96.42	4663/ 96.48	4210/ 96.36	
Visible minority	329/ 3.58	170/ 3.52	159/ 3.64	
**Total resilience score**	6.45 (1.62)	6.60 (1.62)	6.27 (1.60)	8.88 (7604) ***
**Functional resilience score**	7.23 (1.87	7.68 (1.69)	6.71 (1.93)	23.69 (7375) ***
**Psychological resilience score**	5.18 (2.89)	5.11 (2.94)	5.25 (2.83)	−2.18 (8871) *
**Social resilience score**	6.71 (1.90)	6.85 (1.90)	6.56 (1.89)	7.33 (8872) ***
**Personal worry at Baseline wave**				60.53 (1) ***
Less worry	4095/ 52.09	1994/ 47.96	2101/ 56.74	
More worry	3766/ 47.91	2164/ 52.04	1602/ 43.26	
**Comprehensive impact at Baseline wave**				0.65 (1)
Positive	3244/ 38.40	1714/ 38.00	1530/ 38.85	
Negative	5205/ 61.60	2797/ 62.00	2408/ 61.15	
**Comprehensive impact at Exit wave**				0.38 (1) *
Positive	2718/ 35.74	1398/ 34.54	1320/ 37.10	
Negative	4887/ 64.26	2649/ 65.46	2238/ 62.90	

Note: * *p* < 0.05, **, *p* < 0.01, ***, *p* < .001

A supplementary table (Table B) illustrates the associations between the CI (Baseline and Exit waves), PW and the demographic/social-economic backgrounds.

### Logistic regression of MR index on comprehensive impact

The results of the binary logistic regression of CI among our sub-sample of multimorbid participants over age 65 are illustrated in Tables 3 and 4. The key independent variable in Table 3 is the total MRI score. The odds ratios (ORs) for the primary hypotheses represent the change in likelihood of reporting negative self-reported comprehensive impact of the pandemic (versus positive or no impact) for each unit of the MRI or sub-indices (10-point interval scales). ORs under unity represent an inverse association and ORs above unity represent positive associations. We also report 95% confidence intervals (Cis) for the ORs.

**Table 3:** Binary logistic regression for comprehensive impact at COVID-19 survey Baseline and Exit wave (Total multimorbidity resilience score)

	Baseline wave		Exit wave	
	Unadjusted model	Adjusted model	Unadjusted model	Adjusted model
	EXP (B) [95% CI]	EXP (B) [95% CI]	EXP (B) [95% CI]	EXP (B) [95% CI)
**Total multimorbidity resilience score**	0.88 *** [0.86,0.91]	0.83 *** [0.80,0.86]	0.89 *** [0.86,0.92]	0.84 *** [0.81,0.87]
**Sex** (Female)				
Male		1.17 ** [1.05,1.31]		0.88 * [0.79,1.00]
**Age** (65 to 74 years old)				
75 years and older		0.92 [0.83,1.02]		0.87 * [0.78,0.97]
**Marital status** (Not married)				
Married / Common law		1.26 ** [1.07,1.47]		1.26 ** [1.06,1.50]
**Household size** (1 person)				
2 persons		0.95 [0.81,1.13]		0.99 [0.83,1.18]
3 persons and more		0.81 * [0.66,0.99]		0.92 [0.74,1.16]
**Highest education** (University degrees)				
No post-secondary education		0.63 *** [0.54,0.72]		0.59 *** [0.51,0.68]
Post-secondary education		0.68 *** [0.61,0.77]		0.61 *** [0.54,0.70]
**Work status** (Work)				
Non-working		1.03 [0.87,1.23]		1.03 [0.85,1.24]
**Personal income** (Less than $20,000)				
$20,000 to $49,999		1.07 [0.90,1.27]		1.14 [0.95,1.36]
$50,000 to $99,999		1.36 ** [1.14,1.64]		1.35 ** [1.11,1.64]
$100,000 and more		1.57 *** [0.24,2.00]		1.35 * [1.05,1.75]
**Living area** (Rural area)				
Urban area		1.56 *** [1.31,1.85]		1.28 * [1.06,1.54]
**Country of birth** (Canada)				
Out of Canada		1.06 [0.93,1.21]		1.07 [0.93,1.24]
**Ethnicity status** (Not visible minority)				
Visible minority		0.51 *** [0.39,0.68]		0.64 ** [0.48,0.86]
**−2 Log Likelihood**	8958.40	8733.61	7868.27	7709.23
**Cox & Snell R2**	0.01	0.04	0.01	0.03

Note: * *p* < 0.05, **, *p* < 0.01, ***, *p* < .001; Reference group listed in the (−-); Confidence intervals are reported in parentheses under each odds ratio

**Table 4:** Binary logistic regression for comprehensive impact at COVID-19 survey Baseline and Exit wave (three multimorbidity resilience sub-domains scores)

	Baseline wave		Exit wave	
	Unadjusted model	Adjusted model	Unadjusted model	Adjusted model
	EXP (B) [95% CI]	EXP (B) [95% CI]	EXP (B) [95% CI]	EXP (B) [95% CI]
**Functional resilience score**	1.00 [0.97,1.03]	0.97 * [0.94,0.99]	1.02 [0.99,1.05]	0.99 [0.96,1.03]
**Psychological resilience score**	0.90 *** [0.88,0.92]	0.89 *** [0.87,0.91]	0.90 *** [0.88,0.91]	0.89 *** [0.87,0.91]
**Social resilience score**	1.04 ** [1.01,1.08]	1.02 [0.99,1.06]	1.05 ** [1.01,1.08]	1.02 [0.98,1.05]
**Sex** (Female)				
Male		1.19 ** [1.07,1.33]		0.89 [0.79,1.00]
**Age** (65 to 74 years old)				
75 years and older		0.96 [0.97,1.08]		0.94 [0.84,1.06]
**Marital status** (Not married)				
Married / Common law		1.17 [1.00,1.38]		1.19 [1.00,1.41]
**Household size** (1 person)				
2 persons		0.93 [0.79,1.10]		0.97 [0.81,1.16]
3 persons and more		0.79 * [0.64,0.97]		0.90 [0.72,1.12]
**Highest education** (University degrees)				
No post-secondary education		0.64 *** [0.56,0.73]		0.60 *** [0.52,0.69]
Post-secondary education		0.70 *** [0.62,0.79]		0.63 *** [0.55,0.71]
**Work status** (Work)				
Non-working		1.05 [0.88,1.24]		1.04 [0.86,1.25]
**Personal income** (Less than $20,000)				
$20,000 to $49,999		1.06 [0.89,1.25]		1.13 [0.94,1.35]
$50,000 to $99,999		1.36 ** [1.14,1.64]		1.35 ** [1.11,1.64]
$100,000 and more		1.61 *** [1.27,2.05]		1.39 * [1.07,1.79]
**Living area** (Rural area)				
Urban area		1.56 *** [1.31,1.85]		1.28 * [1.06,1.55]
**Country of birth** (Canada)				
Out of Canada		1.08 [0.95,1.23]		1.09 [0.95,1.26]
**Ethnicity status** (Visible minority)				
Non-Visible minority		0.53 *** [0.40,0.70]		0.66 ** [0.49,0.89]
**−2 Log Likelihood**	8906.99	8694.93	7809.11	7670.36
**Cox & Snell R2**	0.02	0.05	0.02	0.04

Note: * *p* < 0.05, **, *p* < 0.01, ***, *p* < .001; Reference group listed in the (−-); Confidence intervals are reported in parentheses under each odds ratio

The total MRI score was inversely associated with CI, in that participants with higher total MR scores were less likely to perceive negative impact of the COVID-19 pandemic at both Baseline wave (OR = 0.83, *p* < 0.001, 95% CI: [0.80,0.86]), and Exit wave (OR = 0.84, *p* < 0.001, 95% CI: [0.81,0.87]), after adjusting for covariates. The associations between demographic and socio-economic covariates and CI were similar at Baseline and Exit wave, except for sex, age and household size. At the Baseline wave, male participants (compared to female) were more likely to report negative comprehensive impact of the COVID-19 pandemic (OR = 1.17, *p* < 0.01, 95% CI: [1.05,1.31]). This relationship reverses at the Exit wave, where male participants were found to be less likely to report negative comprehensive impact of the COVID-19 pandemic than females (OR = 0.88, *p* < 0.05; 95% CI: [0.79,0.99]). Age group is not associated with this outcome at the Baseline wave; however participants who were 75 years and older were less likely to experience negative comprehensive impact of the COVID-19 pandemic than the younger age group (65–74) (OR = 0.87, *p* < 0.05, 95% CI: [0.78,0.98]) at the Exit wave. In addition, participants living with two or more people were less likely to report being negatively affected by the COVID-19 pandemic compared to those living alone (OR = 0.81, *p* < 0.05, 95% CI: [0.66,0.99]) at the Baseline, but not at the Exit wave. Participants who were married or in a common-law relationship, Caucasian, with university degrees, earning more than $50,000 annually (contrast to less than $20,000), and living in an urban area were more likely to report perceived negative impact of the COVID-19 pandemic (see Table 3).

Table 4 explicates the relationships between the three MR sub-index scores and CI. Participants with higher MR-FI were less likely to report a negative impact at the Baseline wave (OR = 0.97, *p* < 0.05, 95% CI: [0.94,0.99]) when all covariates were adjusted, but not at the Exit wave. MR-PI was significantly inversely related to CI at both Baseline (OR = 0.89, *p* < 0.001, 95% CI: [0.87,0.91]) and Exit waves (OR = 0.89, *p* < 0.001, 95% CI: [0.87,0.91]), where participants with higher psychological resilience scores at Follow-up 1 were less likely to be negatively impacted by the COVID-19 pandemic (between April and December 2020). MR-SI was significantly associated with CI at both Baseline and Exit waves in the unadjusted model, but not after the demographic and socio-economic factors were statistically adjusted. The relationships between CI and the demographic and socio-economic factors were similar to the models described in Table 3, except for sex, age and marital status (see Table 4).

We also incorporated a GLMM analysis to model the change of CI from the Baseline to Exit wave. Participants were more likely to report negative (poorer) CI of the COVID-19 pandemic over time. Participants with higher total MRI were less likely to experience negative impact (OR = 0.82, *p* < 0.001, 95% CI: [0.80, 0.85]). Among the three sub-indexes of MRI, MR-PI was the only significant sub-domain in the adjusted model, demonstrating that participants with higher psychological resilience scores were less likely to report negative pandemic comprehensive impact (OR = 0.89, *p* < 0.001, 95% CI: [0.88,0.90]). Sex has both a main effect and interactive effect with the survey wave. Over the course of survey, male participants were less likely to report negative impact compared to female ones (OR = 0.75, *p* < 0.001, 95% CI: [0.64,0.87]). The main effect or interactive effect with the survey wave of age was not supported. Results regarding other covariates were replicated (see Table 5).

**Table 5:** Generalized Linear Mixed Models for comprehensive impact at COVID-19 survey Baseline and Exit waves (Total multimorbidity resilience score and three multimorbidity resilience sub-domains scores)

	Unadjusted model	Adjusted model	Unadjusted model	Adjusted model
	EXP (B) [95% CI]	EXP (B) [95% CI]	EXP (B) [95% CI]	EXP (B) [95% CI]
**Survey wave** (Baseline)				
Exit	1.10 * [1.02,1.18]	1.33 *** [1.17,1.51]	1.10 * [1.02,1.18]	1.33 *** [1.17,1.51]
**Total resilience score**	0.88 *** [0.86,0.90]	0.82 *** [0.80,0.85]		
**Functional resilience score**	–		1.00 [0.98,1.03]	0.98 [0.95,1.00]
**Psychological resilience score**			0.89 *** [0.88,0.91]	0.89 *** [0.88,0.90]
**Social resilience score**			1.05 *** [1.02,1.08]	1.02 [0.99,1.05]
**Sex** (Female)				
Male		1.17 * [1.03,1.31]		1.18 ** [1.05,1.33]
**Sex x Survey wave** (Female)				
Male		0.75 *** [0.64,0.87]		0.75 *** [0.64,0.87]
**Age** (65 to 74 years old)				
75 years and older		0.92 [0.82,1.04]		0.98 [0.87,1.10]
**Age x Survey wave** (65 to 74 years old)				
75 years and older		0.94 [0.80,1.09]		0.94 [0.80,1.10]
**Marital status** (Not married)				
Married / Common law		1.28 ** [1.11,1.47]		1.20 * [1.04,1.38]
**Household size** (1 person)				
2 persons		0.97 [0.84,1.12]		0.94 [0.81,1.09]
3 persons and more		0.87 [0.72,1.04]		0.84 [0.70,1.01]
**Highest education** (University degrees)				
No post-secondary education		0.61 *** [0.54,0.68]		0.62 *** [0.55,0.70]
Post-secondary education		0.65 *** [0.58,0.72]		0.66 *** [0.60,0.74]
**Work status** (Work)				
Non-working		1.03 [0.88,1.20]		1.03 [0.89,1.20]
**Personal income** (Less than $20,000)				
$20,000 to $49,999		1.13 [0.98,1.31]		1.12 [0.97,1.29]
$50,000 to $99,999		1.41 *** [1.21,1.65]		1.41 *** [1.21,1.65]
$100,000 and more		1.53 *** [1.24,1.88]		1.56 *** [1.27,1.92]
**Living area** (Rural area)				
Urban area		1.45 *** [1.24,1.69]		1.45 *** [1.24,1.69]
**Country of birth** (Canada)				
Out of Canada		1.08 [0.96,1.21]		1.09 [0.97,1.23]
**Ethnicity status** (Visible minority)				
Non-Visible minority		1.68 *** [1.31,2.15]		1.63 *** [1.27,2.09]
**AIC**	57,868.23	53,753.11	57,947.95	53,810.80

Note: * *p* < 0.05, **, *p* < 0.01, ***, *p* < .001; Reference group listed in the (−-); Confidence intervals are reported in parentheses under each estimate

### Logistic regression of MR index on worry about COVID-19

The results related to personal worry (PW) about the COVID-19 pandemic are shown in Table 6. In the adjusted models, the participants with higher total MRI scores were less likely to worry about the pandemic (OR = 0.89, *p* < 0.001, 95% CI: [0.86,0.93]). Similarly, participants with higher MR-PI were negatively associated with PW (OR = 0.91, *p* < 0.001, 95% CI: [0.89,0.93]). Participants with higher MR-SI were more likely to report higher levels of PW (OR = 1.04, *p* < 0.05, 95% CI: [1.01,1.07]). No association was found for MR-FI. Also, similar statistically significant relationships between sex, age, income and PW were found in both models with total MRI scores and the three MR sub-index scores. Participants who were male, aged 75 years and older, earning less than $20,000 annually were less likely to worry about the COVID-19 pandemic. Marital status was only significantly related to PW in the model with the total MR score (see Table 6).

**Table 6:** Binary logistic regression for worry at COVID-19 survey Exit wave (Total multimorbidity resilience score and three multimorbidity resilience sub-domains scores)

	Unadjusted model	Adjusted model	Unadjusted model	Adjusted model
	EXP (B) [95% CI]	EXP (B) [95% CI]	EXP (B) [95% CI]	EXP (B) [95% CI]
**Total resilience score**	0.93 *** [0.90,0.96]	0.89 *** [0.86,0.93]	–	
**Functional resilience score**	–		1.03 [1.00,1.06]	1.01 [0.98,1.04]
**Psychological resilience score**			0.91 *** [0.89,0.93]	0.91 *** [0.89,0.93]
**Social resilience score**			1.06 *** [1.03,1.09]	1.04 * [1.01,1.07]
**Sex** (Female)				
Male		0.84 ** [0.75,0.93]		0.84 ** [0.75,0.94]
**Age** (65 to 74 years old)				
75 years and older		0.72 *** [0.65,0.80]		0.77 *** [0.69,0.86]
**Marital status** (Not married)				
Married / Common law		1.23 * [1.05,1.45]		1.16 [0.99,1.37]
**Household size** (1 person)				
2 persons		1.08 [0.91,1.27]		1.05 [0.89,1.24]
3 persons and more		0.96 [0.78,1.18]		0.94 [0.76,1.15]
**Highest education** (University degrees)				
No post-secondary education		0.99 [0.87,1.14]		1.02 [0.89,1.17]
Post-secondary education		1.06 [0.94,1.19]		1.09 [0.96,1.23]
**Work status** (Work)				
Non-working		1.00 [0.84,1.18]		1.00 [0.84,1.19]
**Personal income** (Less than $20,000)				
$20,000 to $49,999		1.20 * [1.01,1.43]		1.19 * [1.01,1.42]
$50,000 to $99,999		1.29 ** [1.07,1.55]		1.29 ** [1.07,1.55]
$100,000 and more		1.35 * [1.07,1.71]		1.38 ** [1.08,1.75]
**Living area** (Rural area)				
Urban area		1.19 [0.99,1.42]		1.19 [1.00,1.43]
**Country of birth** (Canada)				
Out of Canada		0.95 [0.83,1.08]		0.96 [0.84,1.10]
**Ethnicity status** (Visible minority)				
Non-Visible minority		1.07 [0.80,1.42]		1.11 [0.83,1.47]
**−2 Log Likelihood**	8645.24	8561.87	8582.31	8524.74
**Cox & Snell R2**	0.004	0.02	0.01	0.02

Note: * *p* < 0.05, **, *p* < 0.01, ***, *p* < .001; Reference group listed in the (−-); Confidence intervals are reported in parentheses under each odds ratio

## Discussion

One of the most ‘at risk’ groups in the community during the COVID-19 pandemic are those living with multimorbidity, especially the presence of pre-existing respiratory illnesses [49, 50]. Yet not all of these older adults responded to pandemic stressors to the same degree, what we call multimorbidity resilience. This paper examined the effect of a multimorbidity resilience index (total MRI index, and three sub-indexes: functional- MR-FI, psychological (MR-PI), and social – MR-SI) on two pandemic outcomes during 2020: a) perceptions of comprehensive impact; and b) perceptions of worry about COVID-19. We employed three waves of CLSA data (pre-pandemic exposures and peri-pandemic outcomes) to model these effects. This offered a unique opportunity to test the effects of resilience among persons with multimorbidity during the pandemic, which combined to create unparalleled adversity and stress.

At the descriptive level, female multimorbid older participants were slightly more worried about the COVID-19 pandemic than males at the Exit time period September–December 2020); whereas older males felt slightly more negatively impacted at this time than their female counterparts. This might be understood as indicative of perceived vulnerability by sex and age group, which can be heightened when in a partnered relationship (i.e., older males) [21]. We also uncovered age effects, where interestingly, a higher proportion of the younger age group (65–74) were more worried about the COVID-19 pandemic at Baseline and more impacted by the pandemic at Exit compared to the older group (75+). Other research has shown that age differences in pandemic stress and adaptation is not uniform [15].

In testing our primary hypotheses, the multimorbidity resilience index was inversely associated with both pandemic comprehensive impact and personal worry. The associations for the three sub-indexes resulted in distinct associations, depending on the outcome. Although all sub-indexes were inversely associated with comprehensive impact, only the psychological resilience sub-index sustained a statistically significant inverse association once all covariates were adjusted. Additionally, the psychological resilience sub-index was inversely associated with personal worry in the adjusted model; however, unexpectedly the MR social sub-index exhibited a weak positive association with personal worry. The MR functional sub-index revealed modest associations in the hypothesized direction, but not after adjusting for covariates.

Clearly, psychological resilience dominates the overall index effect on these two self-reported pandemic outcomes. The psychological resilience measure combines equal parts of depression, psychological distress, and life satisfaction pre-pandemic. On one level, this might not be surprising since the peri-pandemic outcome measures available were based on self-reports of comprehensive impact on the participants’ lives and perceptions of COVID-19 worry, given the stress-inducing aspects on individual mental health [8, 22, 51, 52]. Additionally, research into the effects of the pandemic on older adults in general suggests that pandemic-induced stress mediation can take place if older individuals proactively pursue effective coping resources at an early stage [15, 16]. The psychological resilience domain encapsulates positive well-being and mental health metrics (levels of depression and distress). Worthy of further examination, qualitative research has revealed several resilience promoting processes including concern for others, community capital, faith, personal experience in dealing with stress in the past, and physical activity [13, 51, 53].

The absence of support (and contrary support for personal worry) for social resilience might be the result of mitigation policies that eroded the protective effect that this domain may have exerted on pandemic stressors. In addition, the weak finding with personal worry that was opposite to our hypothesis could be indicative of concern for others linked to larger social networks. Similarly, the lack of support for the functional domain may also relate to the stay-at-home policies, which reduced the protective effect of this form of resilience on pandemic impact and worry. This suggest that the protective effects of various forms of resilience must be understood relative to unique adversity contexts.

Several covariates were also associated with pandemic comprehensive impact and personal worry. Multimorbid older adults who were the oldest in this sample (75+), living with others, partnered, Caucasian, with higher education, higher incomes, and living in urban areas reported less comprehensive impact of the pandemic. These findings are consistent with research employing a social determinants of health approach to multimorbidity outcomes [21, 38] and formative research on resilience and pandemic and outcomes [2, 3, 12, 49]. Similar relationships were found for personal worry, except those earning less than $20,000 annually were less likely to worry about the COVID-19 pandemic than those with higher incomes, perhaps because they had more to lose due to the pandemic’s effects on the economy.

The implications of this research indicate a need to recognize that not all older adults are equally vulnerable to the stressors inherent in the COVID-19 pandemic. This suggests the need to tailor and target health promotion and public health programs and policies. Among multimorbid older adults, support of interventions that focus attention on depression and distress reduction pre-pandemic and peri-pandemic (e.g., telehealth counselling, community support programs, cognitive therapy, etc.) and enhancement of life satisfaction (healthy aging and activity programs) could foster psychological resilience against future pandemics or other disasters [8, 10, 16]. Clinicians responsible for high risk populations such as multimorbid older adults need to employ interventions early in a pandemic, for instance multidisciplinary rapid response teams [15, 16]. Furthermore, proactive strength-based approaches to enhance psychological forms of resilience, such as positive psychology (mindfulness, meditation, spirituality, dispositional optimism, sleep, etc.), may prove to be valuable forms of preparedness and absorption of pandemic-induced stress as well as peri-pandemic adaptation [6, 14–16, 52]. Clinical interventions require identification of external support systems so that potential resources can be enhanced by fortifying the unique strengths and circumstances of an individual, community or system. Finally, several known mutable social determinants of health also act as protective factors to pandemic impact and worry, in particular income support, literacy, and community support systems pointing to additional fruitful areas of health promotion. The integration of social-psychological resilience models with structural models may prove to be useful to understand pandemic crises, for instance the application of system models of disaster resilience [10], and socio-ecological models of resilience [23], which incorporate multi-level resilience.

A number of limitations need to be recognized. First, the outcome measures are self-report measures reflecting perceptions of comprehensive impact and personal worry about the pandemic. Extension of this research using objective measures may be fruitful. Second, the measures of resilience used in this study were developed specifically from available CLSA data and reflect interactions of resilience and adversity. Other established measures, such as the Connor-Davidson Resilience Score [54], or the Brief Resilient Coping Scale [55], or a priori statistical methods of estimating resilience [56] are worthy of exploration and validation of these results. In addition, there are other resilience measures at the community or system-level (e.g., disaster resilience) that may help to pinpoint socio-ecological areas of import. Third, using a crude indicator of multimorbidity (such as two or more) does not fully capture the type, severity, onset, and unique symptomology. The current study also modelled analyses (not reported in this paper) using three multimorbidity clusters based on subsets of the 27 conditions (cardiovascular; osteo; and a mental health cluster), with replication of the findings. Fourth, the focus on older adults (65+) with multimorbidity limit the generalizability to other groups. Finally, this research needs to be expanded to other groups and populations.

In conclusion, the findings from this study underscore the fact that multimorbid older adults do not experience and respond to multi-level pandemic stressors in the same way. The consistency of psychological forms of resilience point to the importance of fostering public health and health promotion programs and policies that provide the resources and skills to reinforce and maintain this form of multimorbidity resilience during pandemic crises. Future research will undoubtedly extend this work to the larger structural-level system changes that need to be reformed to enhance preparation, absorption and adaption to pandemics.

## Supplementary Information

**Additional file 1.**

## Data Availability

Data are available from the Canadian Longitudinal Study on Aging (www.clsa-elcv.ca) for researchers who meet the criteria for access to de-identified CLSA data.

## Abbreviations

CLSA: Canadian Longitudinal Study on Aging
COVID-19: The Coronavirus Disease-2019
MR: Multimorbidity resilience
MRI: Total MR index
MR-FI: The MR functional sub-index
MR-PI: The MR psychological sub-index
MR-SI: The MR social sub-index
ADL: Activities of Daily Living
IADL: Instrumental Activities of Daily Living
CES-D: Center for Epidemiological Studies Depression
MOS: The total Medical Outcomes Study
LMMR: Lifecourse Model of Multimorbidity Resilience
COVID-B: The COVID Baseline
COVID-E: The COVID Exit
CI: Self-reported comprehensive impact of the COVID-19 pandemic
PW: Self-reported personal worry about COVID-19
GLMM: Generalized Linear Mixed Models
OR: Odds ratios
CI: Confidence intervals
