# Supplementary material for: Multimorbidity resilience and COVID-19 pandemic self-reported impact and worry among older adults: a study based on the Canadian Longitudinal Study on Aging (CLSA)
Source: BMC Geriatr. 2022 Feb 2;22:92. doi: 10.1186/s12877-022-02769-2 (PMC8808267; doi:10.1186/s12877-022-02769-2)
Supplement: Supplementary file 1 — Additional file 1. [file 12877_2022_2769_MOESM1_ESM.docx]

**Supplementary Table A. Inter-correlation Matrix for Multimorbidity Resilience Index (N=9,211)**

|  | Functional resilience | Psychological resilience | Social resilience | Total resilience |
| --- | --- | --- | --- | --- |
| Functional resilience | - | 0.23 *** | 0.18 *** | 0.58 *** |
| Psychological resilience | - | - | 0.47 *** | 0.85 *** |
| Social resilience | - | - | - | 0.73 *** |
| Total resilience | - | - | - | - |
| Note: * p < 0.05, **, p < 0.01, ***, p < .001 | | | | |

**Supplementary Table B. Descriptive data showing all variables by personal worry at Exit wave, and comprehensive impact at Baseline wave and Exit wave (N=9,211)**

|  | **More personal worry**  **at Exit wave**  n/ % | **χ ^2^(df)/t test** | **Negative Comprehensive impact at Baseline wave**  n/ % | **χ ^2^(df)/t test** | **Negative Comprehensive impact at**  **Exit wave**  n/ % | **χ ^2^(df)/t test** |
| --- | --- | --- | --- | --- | --- | --- |
| **Sex**  Female  Male | 2123/ 49.21  1643/ 46.32 | 6.52 (1) * | 2675/ 59.02  2530/ 64.59 | 27.51 (1) *** | 2668/ 64.55  2219/ 63.91 | 0.34 (1) |
| **Age**  65 to 74 years old  75 years and older | 2164/ 52.04  1602/ 43.26 | 60.53 (1) *** | 2979/ 62.00  2408/ 61.15 | 0.65 (1) | 2649/ 65.46  2238/ 62.90 | 5.38 (1) * |
| **Marital status**  Not married  Married / Common law | 1263/ 46.01  2502/ 48.94 | 6.16 (1) * | 1762/ 59.89  3442/ 62.55 | 5.72 (1) * | 1681/ 63.89  3024/ 64.47 | 0.25 (1) |
| **Household size**  1 person  2 persons  3 persons and more | 1119/ 46.18  2179/ 48.90  376/ 47.24 | 4.79 (2) | 1570/ 60.83  2979/ 62.15  541/ 60.99 | 1.41 (2) | 1508/ 64.33  2786/ 64.28  491/ 64.95 | 0.13 (2) |
| **Highest education**  No post-secondary education  Post-secondary education  University degrees | 901/ 52.18  1173/ 48.85  1687/ 47.25 | 1.48 (2) | 1109/ 56.21  1474/ 57.15  2617/ 67.29 | 99.07 (2) *** | 1057/ 58.66  1389/ 60.03  2437/ 69.97 | 92.11 (2) *** |
| **Work status**  Work  Non-working | 356/ 50.28  3350/ 47.69 | 1.74 (1) | 492/ 61.89  4694/ 61.69 | 0.01 (1) | 442/ 64.43  4383/ 64.43 | 0.001 (1) |
| **Personal income**  Less than $20,000  $20,000 to $49,999  $50,000 to $99,999  $100,000 and more | 449/ 45.54  1558/ 47.78  1202/ 48.18  347/ 49.15 | 2.67 (3) | 591/ 55.75  2028/ 58.51  1782/ 65.81  555/ 70.70 | 77.03 (3) | 579/ 60.44  1957/ 62.32  1646/ 67.74  475/ 68.64 | 29.69 (3) *** |
| **Living area**  Rural area  Urban area | 314/ 44.92  3436/ 48.20 | 2.74 (1) | 399/ 52.85  4776/ 62.40 | 26.49 (1) *** | 400/ 59.00  4464/ 64.74 | 8.87 (1) ** |
| **Country of birth**  Canada  Out of Canada | 3067/ 48.53  499/ 45.63 | 4.98 (1) * | 4154/ 61.36  1051/ 62.60 | 0.87 (1) | 3895/ 63.81  992/ 66.09 | 2.72 (1) |
| **Ethnicity status**  Not visible minority  Visible minority | 3631/ 47.90  132/ 48.53 | 0.42 (1) | 5049/ 62.00  152/ 51.18 | 14.20 (2) *** | 4735/ 64.52  147/ 56.98 | 6.17 (1) * |
| Note: * p < 0.05, **, p < 0.01, ***, p < .001 | | | | | | |
